# Lymph node ratio is a prospective prognostic indicator for locally advanced gastric cancer patients after neoadjuvant chemotherapy

**DOI:** 10.1186/s12957-022-02725-9

**Published:** 2022-08-17

**Authors:** Qi Jiang, Xiangyu Zeng, Chenggang Zhang, Ming Yang, Jun Fan, Gan Mao, Qian Shen, Yuping Yin, Weizhen Liu, Kaixiong Tao, Peng Zhang

**Affiliations:** 1 Department of Gastrointestinal Surgery, Union Hospital, Tongji Medical College, Huazhong University of Science and Technology, Wuhan, 430022 Hubei China; 2 Department of Pathology, Union Hospital, Tongji Medical College, Huazhong University of Science and Technology, Wuhan, 430022 Hubei China

**Keywords:** Lymph node ratio, Gastric cancer, Neoadjuvant chemotherapy, Tumor regression grade

## Abstract

**Background:**

The accuracy of lymph node ratio (LNR) as a prognostic index remains to be proven for gastric cancer patients after neoadjuvant chemotherapy (NACT). This study sought to investigate the prognostic value of LNR in locally advanced gastric cancer (LAGC) patients after NACT.

**Methods:**

LAGC patients with clinical TNM stages 2–3, Her2(−), and Eastern Cooperative Oncology Group, scores 0–2 are routinely scheduled with NACT. Patients with LAGC after NACT and surgical operation between January 2012 and October 2020 were retrospectively reviewed. The correlation between LNR and survival was investigated.

**Results:**

Overall, 148 patients were enrolled: 103 with low-LNR (LNR ≤ 30%) and 45 with high-LNR (LNR > 30%). Approximately, 50.5% and 24.4% patients responded to NACT at the primary site in the low-LNR and high-LNR groups, respectively. The overall survival (OS) and progression-free survival (PFS) of low-LNR group were considerably better than those of high-LNR group (3-year OS: 81.9% vs 18.5%, *P* < 0.001; 3-year PFS: 72.6% vs 13.5%, *P* < 0.001). In the low-LNR group, OS and PFS were superior in patients with tumor regression grade (TRG) 0–2 than in those with TRG 3 (3-year OS: 89.2% vs 73.2%, *P* = 0.086; 3-year PFS: 80.3% vs 66.5%, *P* = 0.036). In association with OS and PFS, the degree of tumor differentiation, TRG, and LNR were identified as predictive factors, and LNR was identified as the independent prognostic factor in univariate and multivariate analyses, respectively.

**Conclusions:**

LNR is a prospective index of prognosis in patients with LAGC after NACT.

**Supplementary Information:**

The online version contains supplementary material available at 10.1186/s12957-022-02725-9.

## Introduction

Gastric cancer remains one of the leading diagnosed malignant neoplasms. Globally, the incidence of gastric cancer ranks fourth among all kinds of malignant tumors, and the mortality from it ranked fifth in 2020 [1]. In China, nearly 70% of gastric cancer patients are diagnosed at locally advanced stage with poor prognosis [2]. Therefore, improving the prognosis of these patients is of great significance for improving the overall prognosis of gastric cancer. In recent years, a series of clinical randomized controlled trials such as MAGIC, FLOT, PRODIGY, and RESOLVE have confirmed the efficacy of neoadjuvant chemotherapy (NACT) in the therapy of locally advanced gastric cancer (LAGC) in succession [3–6]. Thus, NACT has been the preferred option for patients with LAGC.

At present, the ypTNM staging system, proposed in the 8th edition of the American Joint Committee on Cancer (AJCC) manual on the basis of the United States National Cancer Database, is the most widely used tool to evaluate the gastric cancer patients’ prognosis after NACT [7]. However, the ypN stage depends on the number of metastasized lymph nodes and its accuracy might be seriously influenced by the dissection or harvesting of insufficient number of lymph nodes. In addition, several investigators have reported that increasing lymph node harvesting has a positive association with better prognosis [8]. Lymph node ratio (LNR, the proportion of metastasized lymph nodes to the dissected lymph nodes) has been confirmed to be a more accurate predictor of prognosis in gastric cancer patients undergoing initial gastrectomy [9–11]. However, there are limited literature on the predictive value of LNR in LAGC patients after NACT [12, 13]. The accuracy of LNR remains to be explored for the patients who underwent NACT. This study sought to further evaluate the prognostic significance of LNR in LAGC patients after NACT.

## Materials and methods

### Patients

The indications for NACT are esophagogastric junction adenocarcinoma with clinical stage T3-4aNanyM0 or non-esophagogastric junction carcinoma with clinical stage T3–4aN+M0 evaluated by CT and endoscopic ultrasonography, Her2(−) detected by immunohistochemistry, and Eastern Cooperative Oncology Group (ECOG) scores 0–2. This retrospective study collected data from LAGC patients who underwent surgery after NACT at the Union Hospital of Tongji Medical College, Huazhong University of Science and Technology, between January 2012 and October 2020. We included the patients on the basis of the following inclusion criteria: (1) gastric cancer confirmed in histological biopsy; (2) clinical TNM stages 2, 3, and 4A; (3) radical gastrectomy combined with D2 lymph node dissection after NACT; and (4) complete clinicopathological data. The exclusion criteria included the following: (1) distant metastasis; (2) gastric stump neoplasms; (3) neoadjuvant therapies in addition to chemotherapy, such as neoadjuvant chemoradiotherapy, NACT combined with targeted therapy, and NACT combined with immunotherapy; and (4) combined with other malignant neoplasms. This research was conducted in line with the Declaration of Helsinki and has been authorized by the Institutional Review Board of Union Hospital of Tongji Medical College.

### Treatments

The main NACT regimens were FOLFOX and SOX, and NACT was generally administered for no less than two cycles. Patients were clinically evaluated by physical conditions, tumor markers, and hematological after every cycle and imaging examination (CT and endoscopic ultrasonography) after two or three cycles, and a multidisciplinary treatment discussion is held to determine further treatment options. If the efficacy evaluation was partial response or stable disease and R0 resection is expected, radical surgery is considered. If the efficacy evaluation was progressive disease and R0 resection cannot be achieved, the systemic treatment is changed. Assessment and documentation of adverse events were on the basis of the Common Terminology Criteria for Adverse Events (CTCAE). For grade 3 or above adverse events, the patients accepted the necessary medical care, including adequate rest, supporting therapy, colony stimulating factors, and even transfusions.

All patients were evaluated by MDT discussions to determine the timing and methods of surgery based on the efficacy of chemotherapy, tumor site, and size. Surgical methods included open or laparoscopic proximal, distal, or total gastrectomy with D2 lymphadenectomy. Pathological response was classified in accordance with the tumor regression grade (TRG) [14]. LNR was the proportion of metastasized lymph nodes to the dissected lymph nodes. The 8th AJCC gastric cancer staging manual was used to evaluate the pathological TNM stage [7]. The assessments of postoperative complications were carried out based on the Clavien-Dindo grading system [15].

The patients with an ECOG scores 0–2 were routinely recommended to receive adjuvant chemotherapy. The oncologist decided the regimens and cycles of adjuvant chemotherapy according to patients’ clinical and pathological reactions.

### Follow-ups

Patients were followed up and assessed every 90 days for the first 24 months, every 180 days for 24 to 60 months, and then yearly after 60 months. We mainly adopt outpatient review and mobile phone to follow up for patients. Follow-up ended in September 2021. The period between the date of operation and the date of death (any cause) was recorded as overall survival (OS). The period between the date of operation and the first observation of progression disease or death (any cause) was recorded as progression-free survival (PFS).

### Statistical analysis

The SPSS software program (26.0 version) was applied for statistical analyses in this research. The normally distributed measurement data was presented in the form of mean ± standard deviation, and the skewed distributed measurement data was reported in the form of median (interquartile range). Frequencies and percentages were employed to describe the categorical variables. The Student *t*-test or Mann-Whitney test was applied to analyze the continuous variables, and the Fisher’s exact test or χ^2^ test was performed to identify the difference in categorical variables appropriately. The clinical data of OS and PFS for the two groups was investigated using the Kaplan-Meier plots coupled with log-rank tests. The prognostic significance of clinicopathological data for OS and PFS was evaluated by utilizing the Cox proportional hazard regression model. The multivariate analysis comprised factors with statistically significant differences in univariate analysis. The tests would be recognized as statistically significant if *P*-values < 0.05.

## Results

### Clinical-pathological characteristics

**Fig. 1:**
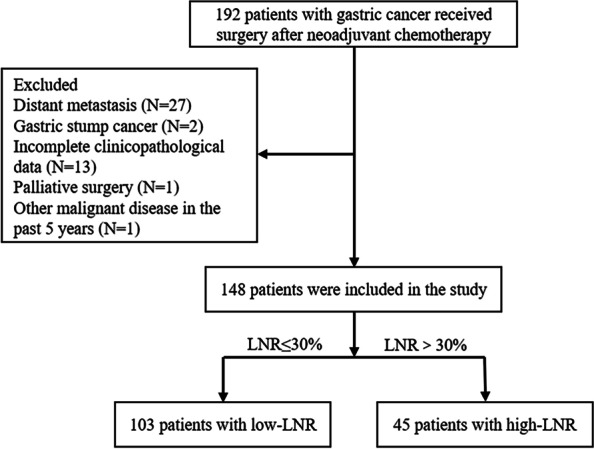
Patients included in this study according to the inclusion and exclusion criteria

A total of 192 gastric cancer patients who received gastrectomy after chemotherapy were enrolled between January 2012 and October 2020. Forty-four patients dropped out for different reasons (Fig. 1). Finally, this research included 148 patients with the median age of 60.0 (range, 52.0–64.8) years, among which 122 (82.4%) were male and 26 (17.6%) were female. Ninety-four (63.5%) patients had tumors situated in the upper stomach, 19 (12.8%) in the middle stomach, and 35 (23.7%) in the lower stomach. Before NACT, 17 (11.5%) patients were diagnosed at clinical stage 2 and 131 (88.5%) at stage 3. The NACT cycle was less than three in 73 (49.3%) patients and no less than three in 75 (50.7%) patients.

As for pathological features, the proportion of tumors with high, moderate, and poor differentiation was 5.4%, 25.0%, and 69.6%, respectively. Five (3.4%) patients acquired TRG 0 grade, and all of them were of pathological complete response; 15 (10.1%), 43 (29.1%), and 85 (57.4%) patients acquired the TRG of 1, 2, and 3 grades, respectively. ypTNM stages 0, 1, 2, 3, and 4 were diagnosed in 5 (3.4%), 19 (12.8%), 40 (27.0%), 74 (50.0%), and 10 (6.8%) cases, respectively.

The total postoperative morbidity was 24.3%. The median hospital stay was 10 (9–12) days. After surgery, 118 (79.7%) patients received adjuvant chemotherapy (Table 1).

**Table 1:** Clinical-pathological characteristics of locally advanced gastric cancer patients with neoadjuvant chemotherapy according to the lymph node ratio

Variables	Total (*n* = 148)	Low-LNR (*n* = 103)	High-LNR (*n* = 45)	*P*
Gender				0.146
Male	122	88 (85.4%)	34 (75.6%)	
Female	26	15 (14.6%)	11 (24.4%)	
Age				0.757
< 65 y	111	78 (75.7%)	33 (73.3%)	
≥ 65 y	37	25 (24.3%)	12 (26.7%)	
Tumor location				0.620
Upper stomach	94	68 (66.0%)	26 (57.8%)	
Middle stomach	19	12 (11.7%)	7 (15.6%)	
Lower stomach	35	23 (22.3%)	12 (26.7%)	
NACT regimen				0.098
FOLFOX	81	51 (49.5%)	30 (66.7%)	
SOX	55	45 (43.7%)	10 (22.2%)	
XELOX	7	4 (3.9%)	3 (6.7%)	
FLOT	5	3 (2.9%)	2 (4.4%)	
NACT cycle				0.174
< 3	73	47 (45.6%)	26 (57.8%)	
≥ 3	75	56 (54.4%)	19 (42.2%)	
Operation method				0.121
Laparoscopic	80	60 (58.3%)	20 (44.4%)	
Open	68	43 (41.7%)	25 (55.6%)	
Resection type				0.226
Proximal	30	23 (22.3%)	7 (15.6%)	
Distal	21	17 (16.5%)	4 (8.9%)	
Total	97	63 (61.2%)	34 (75.6%)	
Number of lymph node harvested		25.3 ± 9.5	21.3 ± 7.4	0.486
Histologic grade				.082
Well	8	7 (6.8%)	1 (2.2%)	
Moderately	37	30 (29.1%)	7 (15.6%)	
Poorly	103	66 (64.1%)	37 (82.2%)	
Margin status				0.287
R0	134	95 (92.2%)	39 (86.7%)	
R1	14	8 (7.8%)	6 (13.3%)	
TRG				.012
0	5	5 (4.9%)	0 (0%)	
1	15	14 (13.6%)	1 (2.2%)	
2	43	33 (32.0%)	10 (22.2%)	
3	85	51 (49.5%)	34 (75.6%)	
ypT stage				.002
0	5	5 (4.9%)	0 (0%)	
1	15	15 (14.6%)	0 (0%)	
2	15	13 (12.6%)	2 (4.4%)	
3	67	46 (44.7%)	21 (46.7%)	
4	46	24 (23.3%)	22 (48.9%)	
Postoperative complications				0.787
Yes	36	25 (24.3%)	10 (22.2%)	
No	113	78 (75.7%)	35 (77.8%)	
Hospital stay				0.201
≤ 12 days	118	85 (82.5%)	33 (73.3%)	
> 12 days	30	18 (17.5%)	12 (26.7%)	
Postoperative chemotherapy				0.696
Yes	118	83 (80.6%)	35 (77.8%)	
No	30	20 (19.4%)	10 (22.2%)	

*Abbreviations*: *LNR* Lymph node ratio, *NACT* Neoadjuvant chemotherapy, *TRG* Tumor regression grade

### Comparing the clinicopathological characteristics between the low and high LNR groups

LNR was calculated for each patient. The optimal cutoff value of LNR was 28.6% according to the receiver operating characteristic analysis. For the convenience of clinical use, we stratified LNR with a 30% boundary. Based on this value, 103 (69.6%) patients were classified as low-LNR (no more than 30% of LNR) and 45 (30.4%) patients as high-LNR (more than 30% of LNR). Comparison of the clinical-pathological characteristics between the two cohorts is presented in Table 1. There was significant difference regarding TRG and ypT stages between the groups (*P* = 0.012 and 0.002, respectively). High-LNR was not related to lower tumor location (*P* = 0.620), less NACT cycle (*P* = 0.174), less lymph nodes harvested (*P* = 0.486), or lower tumor differentiation degree (*P* = 0.082).

Regarding TRG, all patients with TRG 0 grade were in the low-LNR group. Precisely, 52 (50.5%) patients responded to NACT at the primary site (*TRG* = 0, 1, 2) in the low-LNR group, while 11 (24.4%) patients responded in the high-LNR group.

### Comparing the prognosis of the two groups

**Fig. 2:**
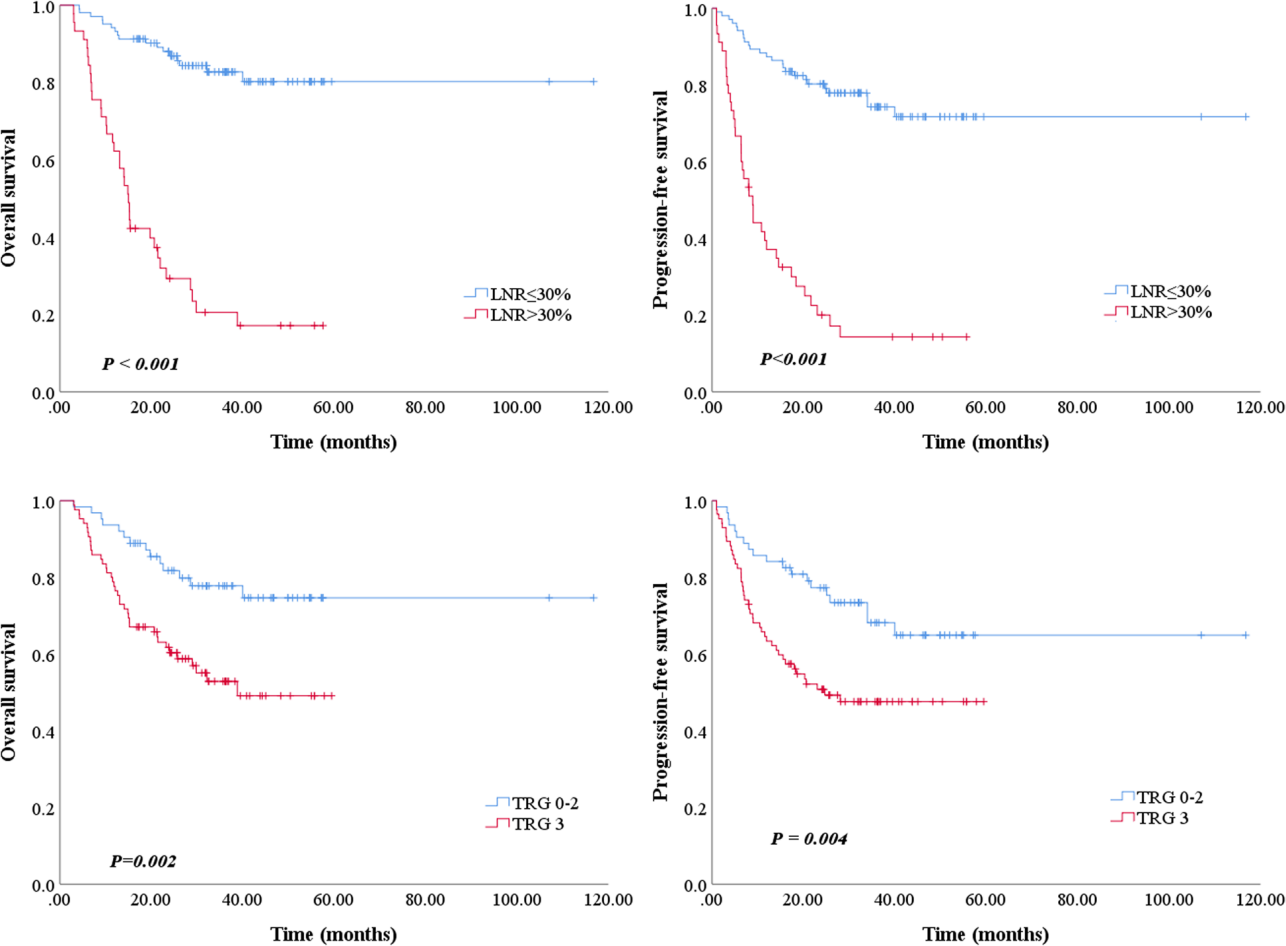
Overall survival and progression-free survival of locally advanced gastric cancer patients with neoadjuvant chemotherapy according to the lymph node ratio and tumor regression grade. LNR, lymph node ratio; TRG, tumor regression grade

The Kaplan-Meier survival curves according to LNR state are showed in Fig. 2. The low-LNR patients got significantly longer OS and PFS than those with high-LNR. The 3-year OS and PFS were 81.9% and 72.6% in the low-LNR group and 18.5% and 13.5% in the high-LNR group (both *P* < 0.001).

**Fig. 3:**
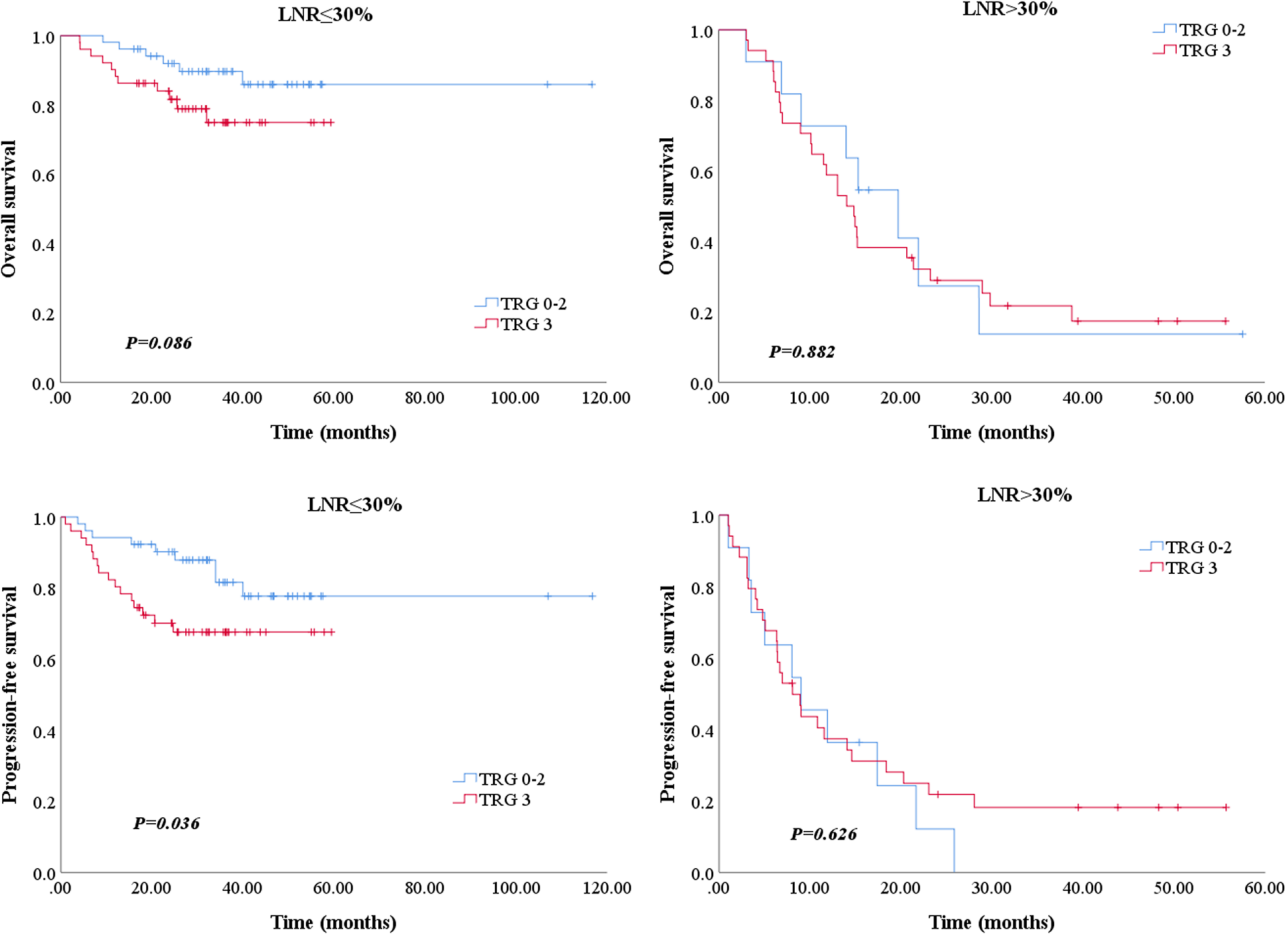
Subgroup analysis of overall survival and progression-free survival in locally advanced gastric cancer patients with LNR ≤ 30% or > 30% according to the tumor regression grade. LNR, lymph node ratio; TRG, tumor regression grade

**Fig. 4:**
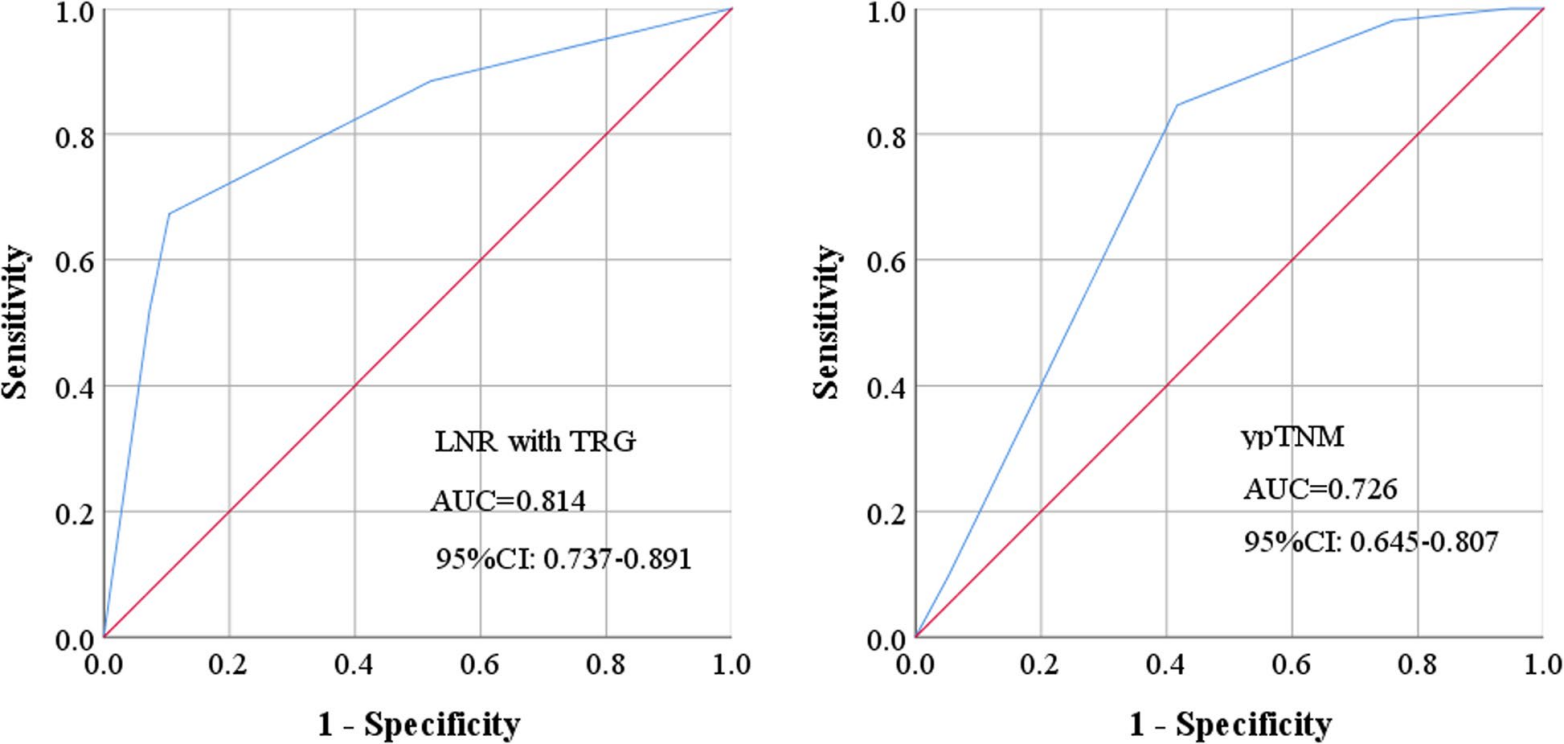
The ROC curves of LNR with TRG and ypTNM stages in predicting death. LNR, lymph node ratio; TRG, tumor regression grade; AUC, area under the curve; CI, confidence interval

Considering the significant difference in tumor response to NACT at the primary site in the two groups, which may affect the prognosis, we included TRG in the prognostic analysis. All patients in the groups were further classified into two cohorts based on TRG: patients who responded to NACT (*TRG* = 0, 1, 2) and nonresponders (*TRG* = 3). Patients who responded to NACT acquired significantly superior OS and PFS than nonresponder. Figure 3 depicts the results of the subgroup analysis. In the low-LNR group, OS was longer in patients who responded to NACT compared with nonresponders (3-year OS: 89.2% vs 73.2%, *P* = 0.086). Patients who responded to NACT also had better PFS than nonresponders (3-year PFS: 80.3% vs 66.5%, *P* = 0.036). While in the high-LNR group, both OS and PFS showed no significant difference between the responders and nonresponders (3-year OS: 12.1% vs 20.0%, *P* = 0.882; 3-year PFS: 0% vs 17.4%, *P* = 0.626). Then, we compared the prognostic efficacy of LNR with TRG and ypTNM stages. When combining LNR with TRG to predict the prognosis, the area under the curve (AUC) in the ROC curve was 0.814 (95% *CI*: 0.737–0.891), which was significantly higher than ypTNM stages (*AUC* = 0.726, 95% *CI*: 0.645–0.807, *P* = 0.007) (Fig. 4).

### Univariate and multivariate analyses

At the last follow-up (September 30, 2021), the median follow-up was 34.6 months. The 1- and 3-year OS was 84.5% and 62.3%, respectively, and the 1- and 3-year PFS was 72.1% and 54.7%, respectively. In the univariate analysis, the degree of differentiation, TRG, and LNR were identified as the predictive factors associated with OS (hazard ratio [HR]: 2.12, 95% confidence interval [CI]: 1.06–4.23, *P* = 0.033; *HR*: 2.58, 95% *CI*: 1.40–4.79, *P* = 0.003; *HR*: 8.21, 95% *CI*: 4.56–14.78, *P* < 0.001) and PFS (*HR*: 1.92, 95% *CI*: 1.04–3.54, *P* = 0.037; *HR*: 2.18, 95% *CI*: 1.27–3.75, *P* = 0.005; *HR*: 6.48, 95% *CI*: 3.86–10.86, *P* < 0.001) (Table 2). However, only LNR was found to be the independent predictive factor for both OS (*HR*: 6.90, 95% *CI*: 3.63–13.14, *P* < 0.001) and PFS (*HR*: 5.58, 95% *CI*: 3.17–9.82, *P* < 0.001) in the multivariate analysis (Table 3).

**Table 2:** Univariate Cox regression analysis of factors associated with survival in locally advanced gastric cancer patients with neoadjuvant chemotherapy

Variables	OS						PFS					
	B	SE	Wald c2	HR	95% *CI*	*P*	B	SE	Wald c2	HR	95% *CI*	*P*
Gender (female vs male)	0.53	0.32	2.73	1.70	0.91–3.19	.099	0.42	0.30	1.86	1.51	0.83–2.75	0.173
Age (< 65 y vs ≥ 65 y)	0.02	0.33	0.00	1.02	0.53–1.94	0.955	0.21	0.31	0.44	1.23	0.67–2.27	0.507
Tumor location (middle/lower vs upper)	0.34	0.28	1.50	1.41	0.81–2.45	0.220	0.23	0.26	0.77	1.26	0.76–2.09	0.379
NACT cycle (< 3 vs ≥ 3)	0.00	0.28	0.00	1.00	0.58–1.73	0.999	0.07	0.25	0.08	1.08	0.65–1.77	0.774
Histologic grade (poorly vs well/moderately)	0.75	0.35	4.54	2.12	1.06–4.23	.033	0.65	0.31	4.35	1.92	1.04–3.54	.037
Margin status (R1 vs R0)	0.72	0.39	3.50	2.06	0.97–4.38	.061	0.67	0.36	3.48	1.96	0.97–3.99	.062
TRG (3 vs 0–2)	0.95	0.31	9.13	2.58	1.40–4.79	.003	0.78	0.28	7.92	2.18	1.27–3.75	.005
Lymph node ratio (> 30% vs ≤ 30%)	2.10	0.30	49.19	8.21	4.56–14.78	< .001	1.87	0.26	50.11	6.48	3.86–10.86	< .001

*Abbreviations*: *OS* Overall survival, *PFS* Progression-free survival, *HR* Hazard ratio, *CI* Confidence interval, *NACT* Neoadjuvant chemotherapy, *TRG* Tumor regression grade

**Table 3:** Multivariate Cox regression analysis of factors associated with survival in locally advanced gastric cancer patients with neoadjuvant chemotherapy

Variables	OS						PFS					
	B	SE	Wald c2	HR	95% *CI*	*P*	B	SE	Wald c2	HR	95% *CI*	*P*
Histologic grade (poorly vs well/moderately)	0.21	0.37	0.31	1.23	0.60–2.54	0.577	0.24	0.32	0.57	1.28	0.68–2.41	0.452
TRG (3 vs 0–2)	0.40	0.33	1.49	1.49	0.78–2.85	0.223	0.30	0.29	1.03	1.34	0.76–2.39	0.311
Lymph node ratio (> 30% vs ≤ 30%)	1.93	0.33	34.59	6.90	3.63–13.14	< .001	1.72	0.29	35.61	5.58	3.17–9.82	< .001

*Abbreviations*: *OS* Overall survival, *PFS* Progression-free survival, *HR* Hazard ratio, *CI* Confidence interval, *NACT* Neoadjuvant chemotherapy, *TRG* Tumor regression grade

## Discussion

NACT followed by surgery can significantly improve the prognosis for gastric cancer patients and has become an alternative standard treatment for LAGC patients. Recently, a series of clinical randomized controlled trials reported that the 3-year PFS in gastric cancer patients underwent NACT coupled with surgery was 59.4–66.3% [3–6, 16]. In our research, the 3-year OS was 62.3%, and the 3-year PFS was 54.7%, which were basically consistent with previous studies. However, the prognosis of gastric cancer patients receiving NACT is highly heterogeneous. Accurate determination of the prognosis is of great significance for the individualized therapy of LAGC patients with NACT.

The pTNM staging system has become a standard forecasting tool of prognosis in gastric cancer patients without neoadjuvant therapy due to its convenience and effectiveness. There is no unified standard prognostic tool for gastric cancer patients undergoing neoadjuvant therapy, and ypTNM staging system is currently the most widely used. However, neoadjuvant therapy is a treatment emerging in recent years. The ypTNM staging system was proposed based on a relatively small number of people, unlike pTNM staging which has been tested and modified in a large sample of people for a long time. The ypT stage can only reflect the depth of tumor invasion at the primary site, but cannot evaluate the effect of tumor response to NACT on prognosis. Neoadjuvant therapy can lead to lymph node regression, and the amount of lymph nodes dissected is prone to be less than 15, which may lead to a large bias when ypN system is used to predict prognosis [17, 18]. Therefore, the accuracy of ypTNM staging in predicting prognosis is insufficient. In this regard, some researchers sought hematology, pathology, and other indicators to supplement ypTNM staging in predicting prognosis. On the other hand, some scholars sought to improve ypTNM staging.

The prognostic indexes of hematology were mainly inflammation and nutrition-related indexes. Previous studies have shown that the increase of inflammatory indicators such as neutrophil-to-lymphocyte ratio, platelet-to-lymphocyte ratio, and lymphocyte to monocyte is significantly negatively associated with the prognosis in gastric cancer patients receiving NACT [19–22]. Furthermore, hemoglobin and prognostic nutritional index are also prognostic predictors of gastric cancer patients after NACT [20, 23, 24]. Pathological indicators include nerve invasion, vascular invasion, and TRG. Blumenthaler et al. found that patients with both lymphovascular invasion and perineural invasion after preoperative treatment for gastric cancer had more severe disease and worse survival outcomes than patients without or with only one of these factors [25]. TRG is defined as the residual tumor accounted for the estimated proportion of the initial tumor in the primary site, which is one of the most important indicators of efficacy evaluation after neoadjuvant therapy [26]. TRG was significantly related with the prognosis in LAGC patients after NACT. A meta-analysis including 3145 patients showed that patients with good response have improved significantly for OS in comparison with those who have no or poor response to neoadjuvant therapy in esophagogastric carcinomas [27]. In our study, low TRG was correlated with significantly melioration in OS and PFS in contrast to high TRG.

The improvement of ypTNM staging focuses on finding alternative indexes for ypT and ypN staging. To improve ypT staging, Tang et al. defined ypTV as π*(tumor diameter/2)^2^*tumor invasion depth and found that ypTV staging was an independent prognostic factor after staging according to cutoff values of ypTV in 253 gastric cancer patients after NACT. The new ypTvNM staging system has better prognostic accuracy than ypTNM [28]. However, this system is complicated and inconvenient for clinical use. Compared with ypN staging, LNR is almost not affected by the number of lymph nodes dissected when judging the prognosis for gastric carcinoma patients after NACT and has been proved to be a more stable and accurate prognostic indicator in previous studies [13, 29, 30]. In the study by Chen et al. (1791), patients obtained from the SEER database were enrolled, and the stratification of LNR instead of ypN staging was used to form a new ypTNrM staging system [12]. In contrast to the ypTNM system, the ypTNrM system could enhance the correct rate of staging and improve the prognostic predictive power. Therefore, in the present study, we classified the patients into two groups based on the cutoff value of LNR. After comparing the prognosis of the two groups, we found that high LNR patients acquired distinctly poorer prognosis comparing to those with low LNR.

Pereira et al. attempted to improve the prognostic prediction effect of gastric cancer patients after NACT by combining lymph node regression and the primary tumor regression [31]. The results showed that the primary tumor regression did not affect the prognosis under the same lymph node regression. Based on the good prognostic prediction effect of LNR, this study combined LNR and TRG to predict the prognosis, and firstly observed that in patients with low LNR, those with good local response to NACT had a better prognosis than patients with poor response. While in patients with high LNR, the response to NACT at the primary site had little effect on the prognosis, which indicated that the biological characteristics of tumor and tumor response are very important for the prognosis of gastric cancer patients after NACT. And when combining LNR with TRG, its diagnostic accuracy in predicting death is better than ypTNM stages. It suggests that LNR with TRG is more responsive to tumor sensitivity to NACT and the effect of NACT and can better excludes the effect of the amount of lymph nodes dissected on prognostic prediction.

In addition, our study found that only LNR stood as the independent predictive indicator in multivariate analysis. Several recent research has also demonstrated that lymph node status, not TRG, was an independent indicator in predicting the prognosis of gastric cancer patients undergoing NACT [32–34]. This suggests that the weight of lymph node status on prognosis is much higher than the primary tumor regression.

Of course, as a retrospective study with a long-term span, the inherent selection bias and the change in the therapy concept of LAGC during this study period cannot be avoided. Secondly, only patients who underwent NACT and radical surgery were included in this study; patients treated with NACT combined with immunotherapy, which has emerged in recent years, were not included; and the influence of neoadjuvant chemotherapy regimen and cycle on prognosis was not evaluated, which may have confounded the analysis of the results. In addition, due to the limited number of patients, we were unable to further validate the findings of this study. A large-scale prospective research is expected to further explore this concept.

## Conclusions

This study demonstrated that LNR is a good predictive index of prognosis in LAGC patients after NACT. High LNR patients acquired distinctly worse prognosis comparing to those with low LNR. In low LNR patients, the prognosis of those with low TRG was worse compared to that of high TRG patients. While in patients with high LNR, TRG had little effect on the prognosis. These findings may have important reference value for the individualized treatment of gastric cancer patients after NACT.

## Supplementary Information

**Additional file 1: Supplementary Figure 1.** The ROC curves of LNR in predicting death. LNR, lymph node ratio; AUC, area under the curve; CI, Confidence interval.

## Data Availability

The datasets supporting the conclusion of this article are included within the article. The underlying datasets are available from the corresponding author on reasonable request.

## Abbreviations

LNR: Lymph node ratio
LAGC: Locally advanced gastric cancer
TRG: Tumor regression grade
NACT: Neoadjuvant chemotherapy
AJCC: American Joint Committee on Cancer
CTCAE: Common Terminology Criteria for Adverse Events
ECOG: Eastern Cooperative Oncology Group
OS: Overall survival
PFS: Progression-free survival
HR: Hazard ratio
CI: Confidence interval
