# Supplementary figures and images for: Lymph node ratio is a prospective prognostic indicator for locally advanced gastric cancer patients after neoadjuvant chemotherapy
Source: World J Surg Oncol. 2022 Aug 17;20:261. doi: 10.1186/s12957-022-02725-9 (PMC9382835; doi:10.1186/s12957-022-02725-9)

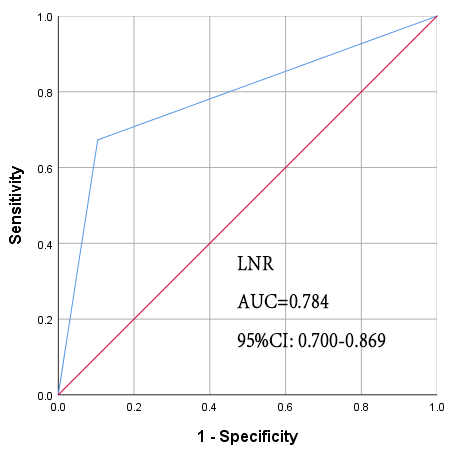

Supplement: Supplementary file 1 — Additional file 1: Supplementary Figure 1. The ROC curves of LNR in predicting death. LNR, lymph node ratio; AUC, area under the curve; CI, Confidence interval. [file 12957_2022_2725_MOESM1_ESM.tif]
